# Synergistic cefiderocol-containing antibiotic combinations active against highly drug-resistant *Acinetobacter baumannii* patient isolates with diverse resistance mechanisms

**DOI:** 10.1093/jac/dkaf306

**Published:** 2025-08-20

**Authors:** Justin Halim, Andrew P Keane, Jeannete Bouzo, Tope Aderibigbe, Jessica A Chicola, Katie T Nolan, Keertana Jonnalagadda, Jason X Tran, Valerie J Carabetta

**Affiliations:** Department of Biomedical Sciences, Cooper Medical School of Rowan University, Camden, NJ 08103, USA; Department of Biomedical Sciences, Cooper Medical School of Rowan University, Camden, NJ 08103, USA; Rowan-Virtua School of Osteopathic Medicine, Stratford, NJ 08084, USA; Department of Biomedical Sciences, Cooper Medical School of Rowan University, Camden, NJ 08103, USA; Department of Chemistry and Biochemistry, Rowan University, Glassboro, NJ 08028, USA; Department of Biomedical Sciences, Cooper Medical School of Rowan University, Camden, NJ 08103, USA; Department of Biomedical Sciences, Cooper Medical School of Rowan University, Camden, NJ 08103, USA; Rowan-Virtua School of Osteopathic Medicine, Stratford, NJ 08084, USA; Rowan-Virtua School of Osteopathic Medicine, Stratford, NJ 08084, USA; Department of Biomedical Sciences, Cooper Medical School of Rowan University, Camden, NJ 08103, USA

## Abstract

**Background:**

*Acinetobacter baumannii* is a nosocomial pathogen known for rapidly developing resistance to nearly all antibiotics, including last-line agents. Cefiderocol, a novel siderophore cephalosporin, has shown *in vitro* activity against *A. baumannii* and is now used clinically, but resistance is emerging. Data on cefiderocol-based antibiotic combinations are limited.

**Objectives:**

To evaluate the *in vitro* activity of cefiderocol alone and in combination with other antibiotics against XDR and PDR *A. baumannii* clinical isolates, and to explore resistance mechanisms underlying cefiderocol synergy.

**Methods:**

We tested 21 XDR/PDR clinical isolates and one NDM-1-producing strain using broth microdilution and checkerboard assays with cefiderocol and 17 antibiotics, including ceftazidime/avibactam, sulbactam/durlobactam, and amikacin. Synergy was determined through checkerboard assays and calculating fractional inhibitory concentration indices (FICI). WGS was used to identify resistance genes in selected strains.

**Results:**

Cefiderocol alone was active against 66.7% of strains and demonstrated synergy with ceftazidime/avibactam and sulbactam/durlobactam in 100% and 95.2% of strains, respectively, and with amikacin, doxycycline and sulbactam in over half of strains. Cefiderocol-based combinations also reduced cefiderocol MICs against an NDM-1-producing strain. WGS revealed β-lactamases and resistance determinants among both susceptible and resistant isolates.

**Conclusions:**

Several cefiderocol-containing combinations show strong *in vitro* synergy against XDR and PDR *A. baumannii*. These combinations, especially cefiderocol-ceftazidime/avibactam and cefiderocol–sulbactam/durlobactam, may enhance treatment of highly resistant *A. baumannii* strains and warrant further clinical investigation.

## Introduction

*Acinetobacter baumannii* is an aerobic, Gram-negative coccobacillus that, while typically of low virulence, is a common cause of hospital-acquired infections, particularly in immunocompromised patients and those undergoing intensive care, mechanical ventilation, or catheterization.^1,2^  *A. baumannii* infections demonstrate high mortality rates, ranging from 40% to 80%.^3,4^ Resistance develops rapidly via acquisition of resistance genes through mobile genetic elements, overproduction of efflux pumps, production of aminoglycoside-modifying enzymes (AMEs) and β-lactamases, and point mutations in target genes such as *gyrA*.^5^ The emergence of multidrug-resistant (MDR), extensively drug-resistant (XDR) and pandrug-resistant (PDR) strains is now a global threat.^5,6^ In particular, carbapenem-resistant *A. baumannii* (CRAB), defined by resistance to imipenem or meropenem, is frequently resistant not only to carbapenems but also to other last-line agents, including colistin and tigecycline.^7–9^ As of 2019, the Center for Disease Control and Prevention (CDC) categorizes CRAB infections as an urgent public health threat.^10^

Cefiderocol, a novel siderophore cephalosporin, was recently approved for hospital- and ventilator-associated pneumonia caused by Gram-negative pathogens.^11^ It structurally resembles ceftazidime and cefepime but includes a catechol moiety that enables iron-mediated uptake, bypassing porins and enhancing intracellular delivery.^12^ Its stability against all Ambler class β-lactamases, including AmpC and ESBLs, further enhances its utility.^12^ Cefiderocol demonstrates strong *in vitro* activity against CRAB and clinical effectiveness in some patients.^13–15^ However, clinical outcomes are mixed. The CREDIBLE-CR trial reported similar infection resolution but higher mortality with cefiderocol compared to the best available therapy.^16^ In contrast, the APEKS-NP trial showed cefiderocol was non-inferior to meropenem for nosocomial pneumonia.^17^ As a result, the IDSA recommends using cefiderocol for CRAB only when alternatives are unavailable and always in combination.^18^ Resistance is increasingly observed, especially among strains expressing OXA-type or metallo-β-lactamases (MBLs), such as NDM.^19^ MBLs are particularly problematic, conferring resistance to nearly all β-lactams.^19^

Synergistic combinations can enhance efficacy and delay resistance.^20^ However, data on cefiderocol-based combinations in *A. baumannii* remain limited. We evaluated the *in vitro* activity of cefiderocol alone and in combination with 17 standard and investigational antibiotics against 21 XDR and PDR *A. baumannii* strains. Previously performed whole-genome sequencing (WGS) showed our strain collection contained diverse β-lactamases but lacked MBLs.^21^ To address this, we included BAA-3302, an NDM-1-harbouring strain. Our goal was to identify synergistic cefiderocol-based combinations and assess β-lactamase-related resistance mechanisms influencing synergy.

## Materials and methods

### Bacterial strains, media and growth conditions

Strains M1–M22 are 21 *A. baumannii* isolates, which are described and characterized in our previous study.^21^ Strain BAA-3302 is a drug-resistant strain that harbours multiple MBLs, including NDM-1, which was purchased from ATCC. Iron-depleted, cation-adjusted Mueller–Hinton broth (ID-CAMHB) was prepared as described previously.^22^ Bacterial strains were inoculated into ID-CAMHB and grown overnight in a 37°C incubator with shaking. Bacterial growth was then assessed by measuring the optical density at 600 nm (OD_600_). Cefiderocol was provided by Shionogi (Florham Park, NJ, USA). Omadacycline was provided by Paratek Pharmaceuticals (Boston, MA, USA). Durlobactam was provided by Innoviva Specialty Therapeutics, Inc. (Waltham, MA, USA). All other antibiotics were purchased as previously described.^23^

### Determination of the MIC by broth microdilution

The MIC values of cefiderocol against each strain were determined using broth microdilution according to standard protocols freely available from the Clinical and Laboratory Standards Institute (CLSI). Following overnight growth, cells were diluted into ID-CAMHB at a starting OD_600_ value of 0.05. Cefiderocol was added to the first well of a flat-bottom 96-well plate at a 2 concentration, with an ‘X’ starting value of 64 mg/L. Two-fold serial dilutions were performed along each row, and an equal volume of diluted bacterial cells was added to each well. The plates were incubated overnight in a 37°C incubator without shaking. The OD_600_ values were read using a Synergy H1 Microplate reader (Biotek) the next day. MIC determinations were made for at least two independent biological replicates. CLSI breakpoint data were used to determine the antimicrobial susceptibility status of the *A. baumannii* strains against cefiderocol. The CLSI considers a MIC of ≤4 to be susceptible, >4 and <16 to be intermediate, and ≥16 to be resistant.^24^ We noted from the checkerboard assays with sulbactam/durlobactam and cefiderocol that the MIC values were 4- to 16-fold lower in ID-CAMHB. Therefore, MIC values were determined for the sulbactam/durlobactam in both CAMHB and ID-CAMHB to guide appropriate ‘X’ starting values for sulbactam/durlobactam-cefiderocol checkerboard assays.

### Screening for combinatorial effects by disc stacking assays

Disc stacking assays were performed to screen for potential synergistic or additive interactions between cefiderocol and other antibiotics, as previously described.^25^ Overnight cultures were diluted into 2 mL of fresh ID-CAMHB and allowed to grow at 37°C for 2–3 h. We selected strain M9, a PDR strain, as a representative strain for these disc stacking assays. Following this pre-growth phase, 300 μL of culture was spread onto a 150 mm × 15 mm MHA plate. Commercially available discs containing tetracycline, trimethoprim-sulfamethoxazole, levofloxacin, ceftriaxone and piperacillin-tazobactam were used. For all other antibiotics tested (cefiderocol, meropenem, ampicillin/sulbactam, sulbactam, amikacin, minocycline, tigecycline and rifampin), filter paper discs 6 mm in diameter were prepared by adding a specific amount of drug according to CLSI recommendations, as previously described, and allowing them to dry for at least 1 h at 30°C.^23^ Plates were prepared, evaluating cefiderocol in combination with each of the antibiotics. Four antibiotic discs, two impregnated with cefiderocol and two impregnated with a different antibiotic, were placed on the surface of the agar, evenly spaced from each other. A disc impregnated with cefiderocol was then placed on top of one of the discs containing the other antibiotic, and a disc impregnated with the other antibiotic was placed on top of one of the cefiderocol discs. 30 μL of sterile phosphate-buffered saline was added to the top of the stacked discs. Plates were then incubated overnight in a 37°C incubator, and the zones of inhibition were measured the following day. A larger zone of inhibition of >3 mm surrounding the stacked discs compared to each drug alone indicated a potential synergistic or additive interaction, which was then further evaluated by the checkerboard assay.

### Checkerboard assays

Checkerboard assays were performed as previously described.^23^ Two-fold serial dilutions of cefiderocol and a different antibiotic were performed horizontally and vertically in a 96-well plate. Antibiotics were initially added at a 4× concentration, using the same ‘×’ starting values as previously described.^23^ For checkerboard assays involving sulbactam/durlobactam, sulbactam was added at an initial 4× concentration of 32 g/L, and serial dilutions were then performed, while durlobactam was added at a constant concentration of 4 g/L per well, per CLSI recommendations.^24^ Similarly, for checkerboard assays involving ceftazidime/avibactam, ceftazidime was added at an initial 4× concentration with subsequent serial dilutions performed, while avibactam was added at a constant concentration of 4 mg/L per well.^24^ Diluted cells were then added at an OD_600_ of 0.05 and incubated overnight. The OD_600_ was read the following morning. Combinatorial effects were measured by calculating the fractional inhibitory concentration index (FICI). The fractional inhibitory concentration (FIC) for each drug was determined, which is the concentration of the antibiotic in combination divided by the MIC of the antibiotic alone. The FICI was then calculated using the following formula: FICI = (MIC A_A_ _+_ _B_/MIC A) + (MIC B_A_ _+_ _B_/MIC B), in which MIC A and MIC B denote the MIC value of each antibiotic alone and MIC A_A_ _+_ _B_ and MIC B_A_ _+_ _B_ denote the MIC values of the drugs in combination. For a FICI of ≤0.5, the combination is synergistic; for a FICI of 0.5–1.0, the combination is additive; for a FICI >1 and <4, there is no effect; for a FICI ≥4, the interaction is antagonistic. All determinations were made at least two independent times.

## Results

### Determination of susceptibilities of *A. baumannii* clinical isolates to cefiderocol

We evaluated the susceptibility of 21 previously characterized XDR and PDR *A. baumannii* isolates (strains M1–M22) to cefiderocol via broth microdilution (Table 1).^21^ Fourteen strains (66.7%) were susceptible (MIC ≤ 4 mg/L), three (14.3%) were intermediate, and four (19.0%) were resistant (MIC ≥ 16 mg/L). Non-susceptible strains, defined as intermediate or resistant, included M1, M3, M4, M5, M6, M8 and M22. An additional MBL-harbouring strain, BAA-3302, was also resistant (MIC = 16 mg/L).

**Table 1.** Antibiotic susceptibilities of each *A. baumannii* isolate to cefiderocol

Strain	MIC 1	MIC 2	S/I/R	Strain	MIC 1	MIC 2	S/I/R
M1	64	64	R	M12	2	2	S
M2	1	1	S	M13	0.5	1	S
M3	32	64	R	M14	0.5	1	S
M4	8	16	I	M16	1	1	S
M5	16	16	R	M17	0.5	1	S
M6	32	32	R	M18	0.5	1	S
M7	0.5	1	S	M19	1	1	S
M8	8	16	I	M20	1	2	S
M9	1	2	S	M21	1	1	S
M10	1	1	S	M22	8	16	I
M11	2	4	S	BAA3302	16	16	R

Strains are labelled M1–M22.

Note that strain M15 was excluded from the study, as it was not *A. baumannii*. MIC values were determined at least two independent times, with the determinations listed as MIC 1 and MIC 2.

Strains that are susceptible are denoted as S; strains with intermediate susceptibility are denoted as I; strains that are resistant are denoted as R, according to CLSI standards.

### Determination of the combinatorial effects of cefiderocol with various antibiotics

**Figure 1.**
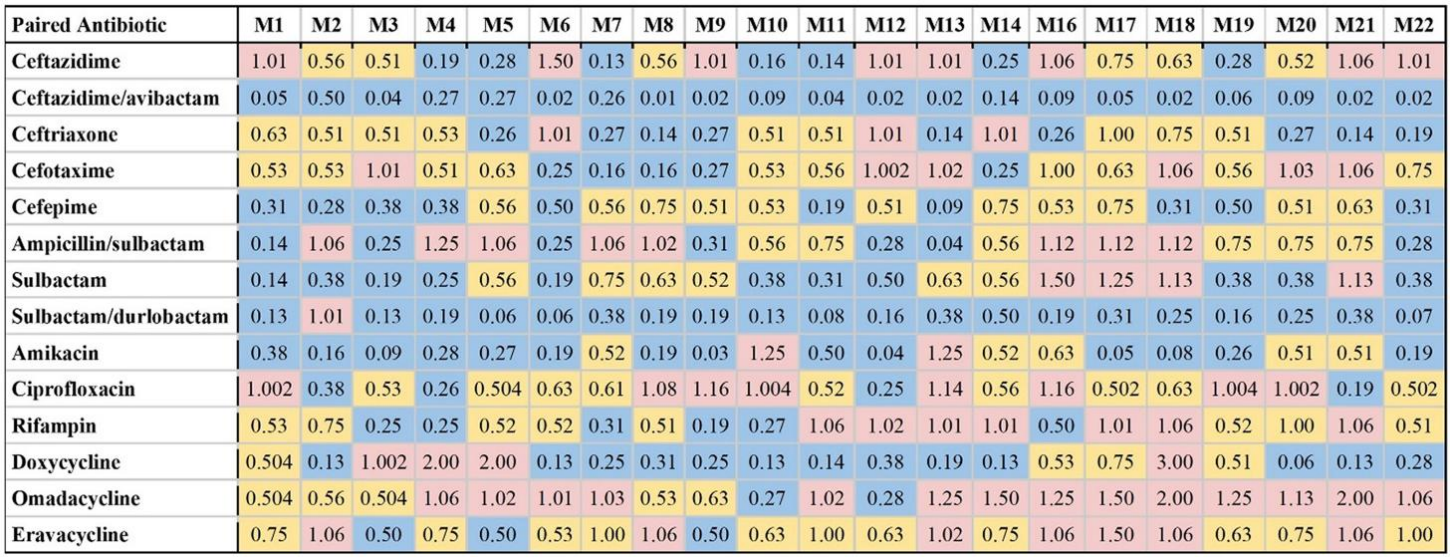
Fractional inhibitory concentration index (FICI) values obtained from cefiderocol in combination with various antibiotics against *A. baumannii* strains M1–M22. FICI values in the synergistic range (≤0.5) are reported in blue; FICI values in the additive range (0.5–1.0) are reported in yellow; FICI values indicating no interaction (1.0–4.0) are reported in pink. FICI values indicating no interaction (1.0–4.0) are reported in pink.

We initially screened 17 cefiderocol-antibiotic combinations against the PDR strain M9 using disc stacking. Eleven combinations showing ≥3 mm increased inhibition zones were further evaluated via checkerboard assays (Table S1, available as Supplementary data at *JAC* Online). We also tested cefiderocol with sulbactam/durlobactam, eravacycline and omadacycline, which have shown *in vitro* activity against *A. baumannii* (Table S2).^26–28^ No antagonistic interactions were found. FICI values were calculated to categorize interactions (Figure 1), and the frequency of synergy, additive effects, or indifference is summarized in Table 2.

**Table 2.** Rates of synergistic, additive, or lack of effects between antibiotics paired with cefiderocol against strains M1–M22

Paired Antibiotic	Synergistic	Additive	Indifferent
Ceftazidime	33.3%	28.6%	38.1%
Ceftazidime/avibactam	100.0%	0.0%	0.0%
Ceftriaxone	42.9%	42.9%	14.3%
Cefotaxime	23.8%	47.6%	28.6%
Cefepime	47.6%	52.4%	0.0%
Ampicillin/sulbactam	38.1%	28.6%	33.3%
Sulbactam	52.4%	28.6%	19.0%
Sulbactam/durlobactam	95.2%	0.0%	4.8%
Amikacin	66.7%	23.8%	9.5%
Ciprofloxacin	19.0%	42.9%	38.1%
Rifampin	23.8%	42.9%	33.3%
Doxycycline	61.9%	19.0%	19.0%
Omadacycline	9.5%	19.0%	71.4%
Eravacycline	14.3%	52.4%	33.3%

**Figure 2.**
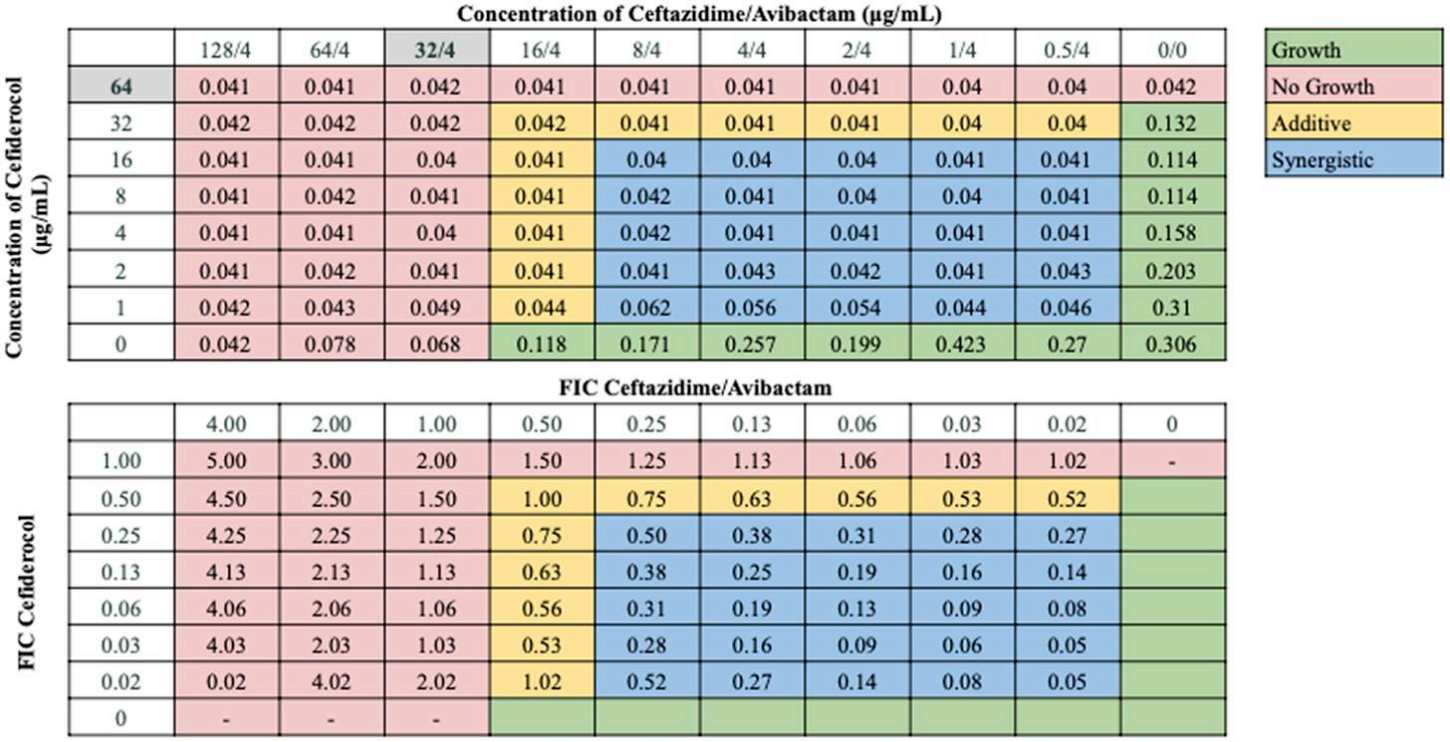
Representative checkerboard assay with cefiderocol and ceftazidime/avibactam against strain M1. Top: OD_600_ measurements following 16 h of static growth at 37°C. The MIC value for ceftazidime/avibactam alone is highlighted. There is no highlighted MIC value for cefiderocol as the MIC exceeded the maximum starting concentration. No bacterial growth occurred in wells where OD_600_ < 0.1, and above this cutoff, bacterial growth did occur. Bottom: FIC values were calculated for each drug (concentration/MIC) and added together for all wells where no growth was observed. Additive interactions (FICI between 0.5 and 1.0), and synergistic interactions (FICI ≤ 0.5) are indicated.

**Figure 3.**
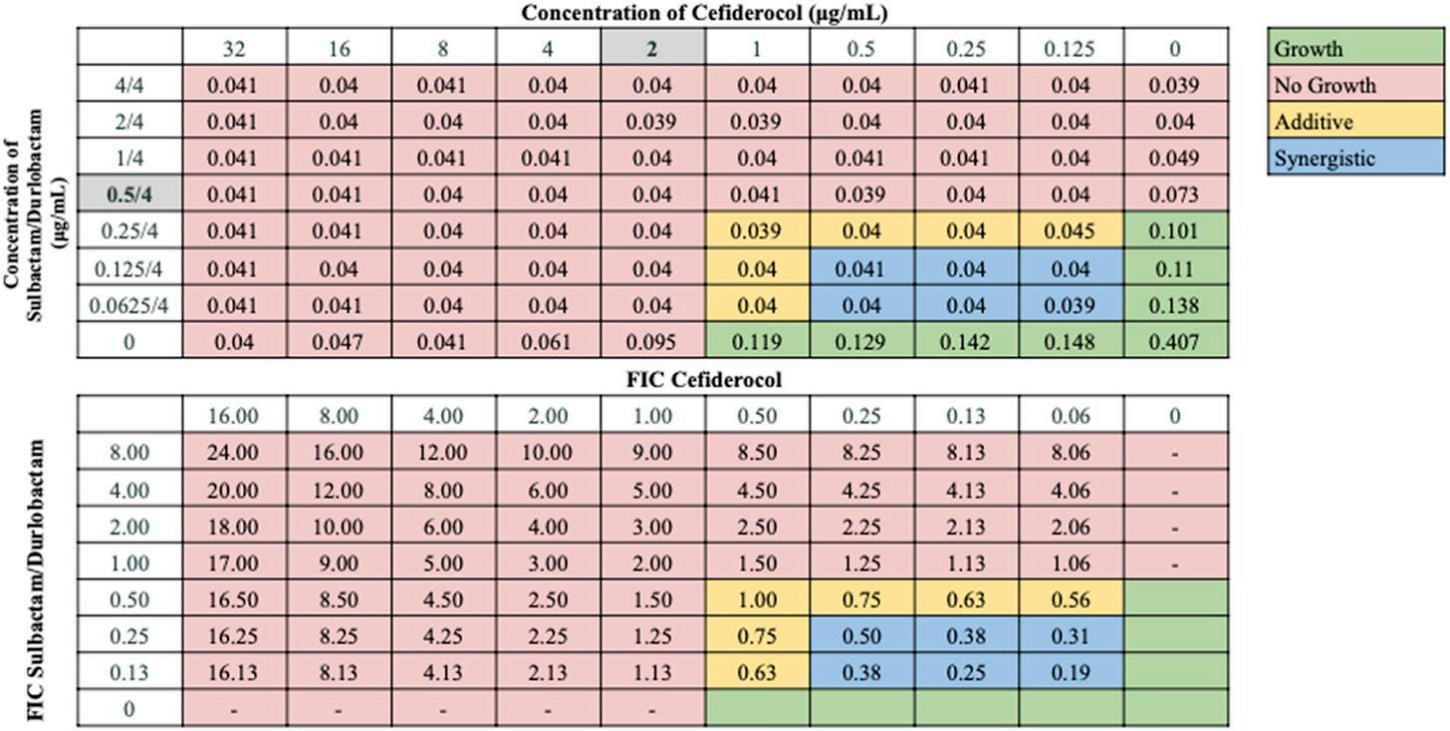
Representative checkerboard assay with cefiderocol and sulbactam/durlobactam against strain M16. Top: OD_600_ measurements following 16 h of static growth at 37°C. The MIC values for each drug alone are highlighted. No bacterial growth occurred in wells where OD_600_ < 0.1, and above this cutoff, bacterial growth did occur. Bottom: FIC values were calculated for each drug (concentration/MIC) and added together for all wells where no growth was observed. Additive interactions (FICI between 0.5 and 1.0), and synergistic interactions (FICI ≤ 0.5) are indicated.

**Figure 4.**
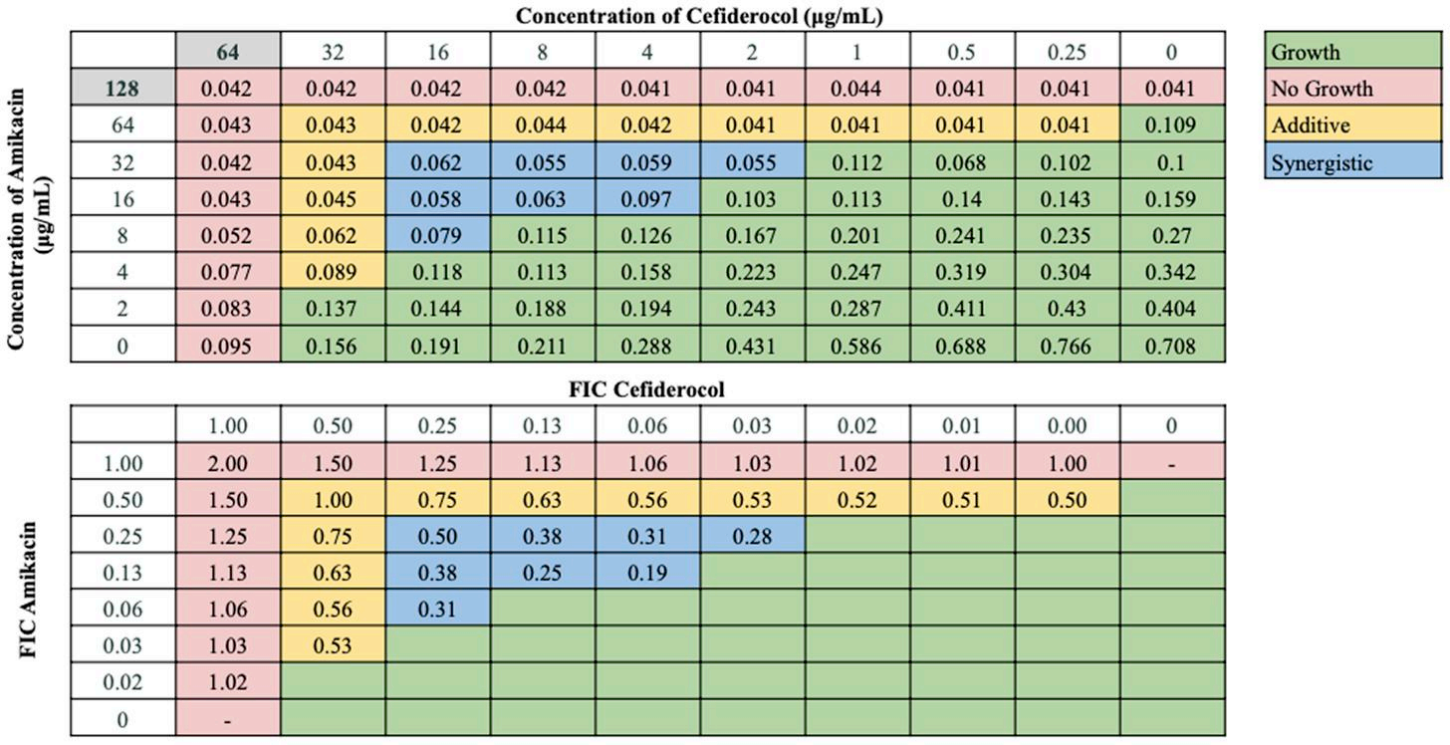
Representative checkerboard assay with cefiderocol and amikacin against strain M6. Top: OD_600_ measurements following 16 h of static growth at 37°C. The MIC values for each drug alone are highlighted. No bacterial growth occurred in wells here OD_600_ < 0.1, and above this cutoff, bacterial growth did occur. Bottom: FIC values were calculated for each drug (concentration/MIC) and added together for all wells where no growth was observed. Additive interactions (FICI between 0.5 and 1.0), and synergistic interactions (FICI ≤ 0.5) are indicated.

**Figure 5.**
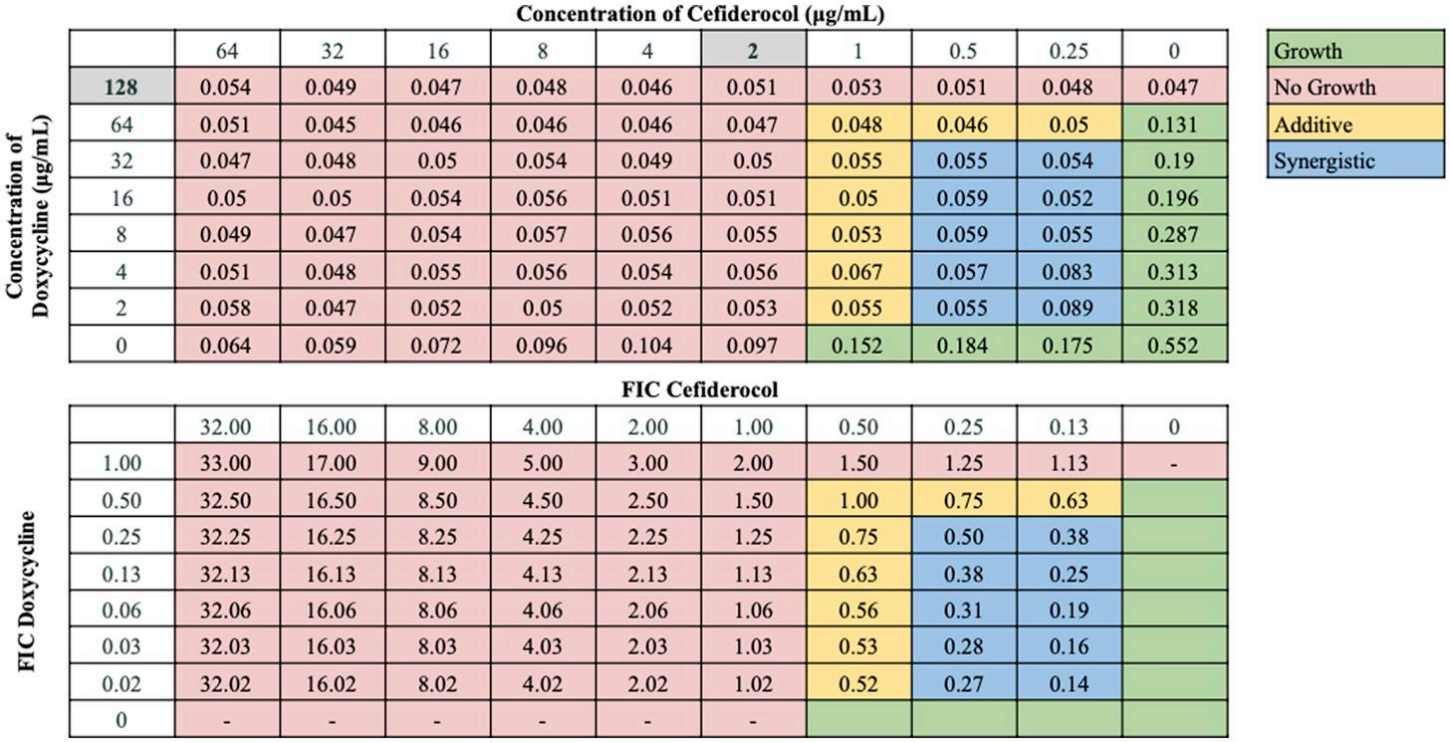
Representative checkerboard assay with cefiderocol and doxycycline against strain M11. Top: OD_600_ measurements following 16 h of static growth at 37°C. The MIC values for each drug alone are highlighted. No bacterial growth occurred in wells where OD_600_ < 0.1, and above this cutoff, bacterial growth did occur. Bottom: FIC values were calculated for each drug (concentration/MIC) and added together for all wells where no growth was observed. Additive interactions (FICI between 0.5 and 1.0), and synergistic interactions (FICI ≤ 0.5) are indicated.

Interactions between antibiotic pairings were not uniform for all strains tested, in agreement with our previous findings.^21^ Several cefiderocol-based combinations displayed synergy or additive effects across the 21-strain panel. Notably, cefiderocol demonstrated the highest synergy rates in combination with ceftazidime/avibactam (100.0%) and sulbactam/durlobactam (95.2%). Cefiderocol and amikacin showed synergy against 66.7% of strains, including all strains non-susceptible to cefiderocol alone, and additive effects against 23.8% of strains. Doxycycline and cefiderocol showed synergy against 61.9% of strains, and additive effects against 19.0% of strains. Sulbactam and cefiderocol were also relatively effective, demonstrating synergy against 52.4% of strains, and additive effects against 28.6% of strains. cefiderocol combined with other β-lactam drugs, including cefepime, ceftriaxone, cefotaxime and ampicillin–sulbactam, showed moderate synergy rates ranging from 23.8 to 52.4%. Rifampin and ciprofloxacin displayed synergy in 19.0% and 23.8% of strains in combination with cefiderocol, respectively, and additive effects against nearly half of the strains. In contrast, cefiderocol paired with omadacycline or eravacycline showed limited synergy (<15%), despite these drugs’ efficacy as monotherapy,^21^ although eravacycline and cefiderocol showed additive effects against 52.4% of strains. No antagonism was observed with any combination. Representative checkerboard assays displaying synergy patterns are shown for four combinations (Figures 2–5), including cefiderocol paired with ceftazidime/avibactam, sulbactam/durlobactam, amikacin and doxycycline. Representative checkerboard assays demonstrating synergy patterns for cefiderocol combined with eravacycline, omadacycline, rifampin, ciprofloxacin, ceftazidime, cefepime, ceftriaxone, cefotaxime, ampicillin/sulbactam and sulbactam are shown in Figures S1–S10.

### Identification of resistance genes among cefiderocol-resistant *A. baumannii* isolates

Previously, WGS was performed on eight selected strains, including three that were non-susceptible to cefiderocol (M1, M4 and M5) and five that were susceptible (M9, M10, M11, M13 and M20).^21^ Resistance gene profiles are summarized in Table 3. All harboured class C ADC-1-like β-lactamases, such as *blaADC-30, blaADC-33, blaADC-73, blaADC-80* and *blaADC-150*. ADC-33 and ADC-73 appeared in both groups, while ADC-30, ADC-80 and ADC-150 were exclusive to susceptible strains. All strains also harboured class D (OXA) β-lactamases, including the acquired genes *blaOXA-23* and *blaOXA-421,* as well as intrinsic OXA-51-like alleles *blaOXA-66, blaOXA-83* and *blaOXA-94.* OXA-82 was detected only in M1, an FDC-resistant strain. OXA-23 and OXA-66 were found across many sequenced isolates. OXA-94 was present in one non-susceptible and one susceptible strain. OXA-421 was limited to susceptible strains. Class B (MBL) β-lactamases were not found in our original collection of strains. A mutation in *ftsI* (PBP3) was also identified in both susceptible and non-susceptible isolates, suggesting that this is not a major determinant of cefiderocol resistance. All strains carried aminoglycoside resistance determinants, including AME genes and *armA*, as well as *tetB* for tetracycline resistance and *gyrA* mutations linked to fluoroquinolone resistance.

**Table 3.** Identified resistance genes among nine selected *A. baumannii* strains

Cefiderocol Susceptibility	Strain	MIC	β-Lactam Resistance	Other Resistance
Non-susceptible	M1	64	*blaADC-33, blaOXA-23,* *blaOXA-82*	*aac(3)-Ia, aadA1, aph(3’)-Via, aph(6)-Id, ant(3’)-IIa, armA, tet(B), gyrA_S81L, parC_S84L, mph(E), msr(E), sul1, sul2*
	M4	8, 16	*blaADC-73, blaOXA-23,* *blaOXA-66, fts1_A515V*	*aph(3’)-Ib, aph(6)-Id, ant(3’)-IIa, armA, tet(B), gyrA_S81L, parC_S84L, mph(E), msr(E), sul2*
	M5	16	*blaADC-73, blaOXA-23,* *blaOXA-66, fts1_A515V*	*aph(3’)-Ib, aph(6)-Id, ant(3’)-IIa, armA, tet(B), gyrA_S81L, parC_S84L, mph(E), msr(E), sul2*
	BAA-3302	16	*blaNDM-1, blaL1, blaOXA-94*	*adeABC, adeFGH, adeIJK, adeL, adeN, adeRS, baeSR, ant2-Ia, ant(3’)-II, aph3-VI, catB, emrB, macAB, tolC, mphD, msbA, msrE, rlmN, soxR, sul2*
Susceptible	M9	1, 2	*blaADC-33, blaOXA-66, fts1_A515V*	*aadA1, aph(3’)-Ib, aph(6)-Id, ant(3’)-IIa, armA, tet(B), gyrA_S81L, parC_S84L, mph(E), msr(E), sul1, sul2*
	M10	1	*blaADC-30, blaOXA-66*	*aac(3)-Ia, aac(6’)-Ib’, aadA1, aph(3’)-Ib, aph(3’)-Ia, aph(6)_Id, ant(3’)-IIa, tet(B), gyrA_S81L, parC_S84L, mph(E), catB8, sul1, sul2*
	M11	2, 4	*blaADC-80, blaOXA-94*	*aph(3’)-Ib, aph(6)-Id, ant(3’)-IIa, tet(B), gyrA_S81L, parC_E88K, floR, sul1, sul2*
	M13	0.5, 1	*blaADC-150, blaOXA-66,* *blaOXA-421*	*aac(3)-Ia, aadA1, aph(3’)-Ib, aph(6)_Id, ant(3’)-IIa, tet(B), parC_S84L, sul1, sul2*
	M20	1, 2	*blaADC-73, blaOXA-23,* *blaOXA-66, fts1_A515V*	*aadA1, aph(3’)-Ib, aph(6)_Id, ant(3’)-IIa, armA, tet(B), gyrA_S81L, parC_S84L, mph(E), msr(E), sul1, sul2*

Strains with cefiderocol MIC values >4 are categorized as non-susceptible, while those with MIC values ≤4 are categorized as susceptible.

Sequencing data are available on the GeoSeeq platform, a publicly accessible database connecting researchers with tools for public health surveillance (https://portal.geoseeq.com/sample-groups/63d25b1e-8039-4b1d-bbb8-ab461d436055).

### Cefiderocol-based combinations against an NDM-1-producing *A. baumannii* isolate

**Figure 6.**
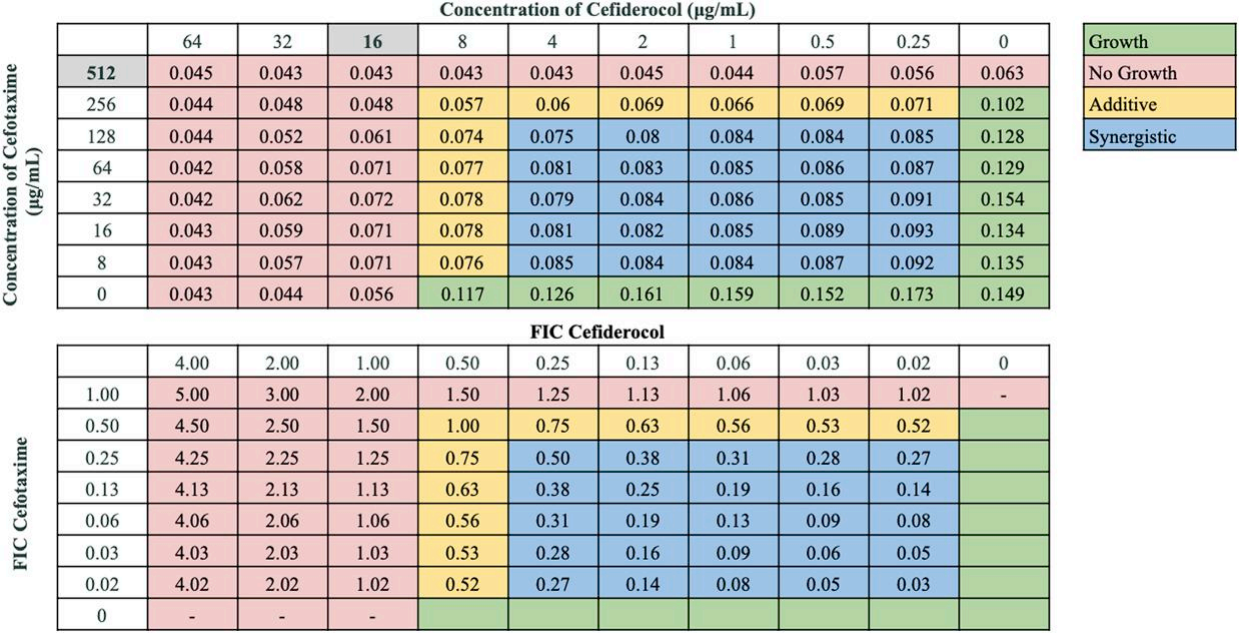
Representative checkerboard assay with cefiderocol and cefotaxime against strain BAA-3302. Top: OD_600_ measurements following 16 h of static growth at 37°C. The MIC values for each drug alone are highlighted. No bacterial growth occurred in wells where OD_600_ < 0.1, and above this cutoff, bacterial growth did occur. Bottom: FIC values were calculated for each drug (concentration/MIC) and added together for all wells where no growth was observed. Additive interactions (FICI between 0.5 and 1.0), and synergistic interactions (FICI ≤ 0.5) are indicated.

Cefiderocol alone was ineffective against MBL-harbouring BAA-3302 (MIC = 16 mg/L). However, combining cefiderocol with cephalosporins (ceftazidime, ceftriaxone, cefotaxime and cefepime) or β-lactam/β-lactam inhibitors (ampicillin/sulbactam, ceftazidime/avibactam and sulbactam/durlobactam) reduced MICs to ≤1 mg/L (Table 4). Amikacin, ciprofloxacin and rifampin also demonstrated synergy. Tetracyclines showed no synergy despite baseline susceptibility, possibly due to the presence of resistance-nodulation-division (RND)-type efflux pumps (*adeABC*, *adeFGH* and *adeIJK*). Representative checkerboards for cefotaxime and ciprofloxacin are shown in Figures 6 and S11.

**Table 4.** Combinatorial effects of cefiderocol-containing antibiotic combinations against strain BAA-3302, an NDM-1-harbouring strain resistant to cefiderocol (MIC = 16 mg/L)

Paired antibiotic	FICI Value	Combined cefiderocol MIC (mg/L)	Interpretation
Ceftazidime	0.19	0.25	Synergy
Ceftazidime/avibactam	0.19	1	Synergy
Ceftriaxone	0.05	0.25	Synergy
Cefotaxime	0.03	0.25	Synergy
Cefepime	0.27	0.25	Synergy
Ampicillin/sulbactam	0.14	0.25	Synergy
Sulbactam	1.01	16	No interaction
Sulbactam/durlobactam	0.19	1	Synergy
Amikacin	0.27	0.25	Synergy
Ciprofloxacin	0.03	0.25	Synergy
Rifampin	0.08	0.25	Synergy
Doxycycline	1.03	16	No interaction
Omadacycline	1.03	16	No interaction
Eravacycline	1.25	16	No interaction

FICI values are reported, as well as MIC values of cefiderocol in the presence of each paired antibiotic.

## Discussion

### Cefiderocol activity and resistance mechanisms

As multidrug resistance rises in *A. baumannii*, treatment options become more limited. Cefiderocol has demonstrated *in vitro* activity against CRAB and is used clinically, though with variable outcomes.^13–15^ Cefiderocol features a catechol moiety, which enables iron-mediated uptake via siderophore receptors and bypasses porins, a mechanism commonly referred to as the ‘Trojan Horse’ mechanism.^11,12,29^ Cefiderocol’s unique mechanism and large structure confer increased stability against common antibiotic resistance mechanisms, particularly β-lactamases of all Ambler classes.^29,30^ In our panel of 21 XDR and PDR strains, 66.7% were susceptible to cefiderocol. Resistance was limited to four isolates (M1, M3, M5 and M6) and the MBL-producing strain BAA-3302. Known resistance mechanisms include overexpression of multiple β-lactamases (NDM, KPC, OXA-type and AmpC variants) and impaired siderophore uptake via TonB-related PiuA/PirA loss.^19,31^

WGS revealed a range of β-lactamases in both susceptible and non-susceptible strains. M1, with the highest MIC, was the only strain harbouring *blaOXA-82*, suggesting a role in resistance. OXA-82, an OXA-51-like variant, is intrinsic to *A. baumannii* and is generally insufficient to confer high-level resistance unless upregulated by insertion sequences such as ISAba1 or ISAba125, which we did not evaluate.^32,33^ To further analyse the potential role of these β-lactamases in cefiderocol resistance, we will build IS element detection into our bioinformatic pipeline. Other OXA-51-like variants, OXA-66, and OXA-94, were widely distributed among cefiderocol-susceptible strains and likely insufficient alone to confer resistance. OXA-23, an acquired carbapenemase, was identified in three non-susceptible strains and one susceptible strain.^32^ OXA-421, an acquired OXA-270-like carbapenemase typically found in *Acinetobacter pitti*, was found only in a susceptible strain and appears non-contributory.^32^ Class C ADC-1-like cephalosporinases (e.g. ADC-33, ADC-73, and ADC-80) occurred in both groups. Similarly, ADC-1-like cephalosporinases are also intrinsic to *A. baumannii* and likely confer minimal resistance, though they may contribute to higher level resistance when co-expressed with OXA enzymes.^32^ PBP3 mutations were also observed across groups, suggesting limited relevance. BAA-3302 carried *blaNDM-1, blaL1* and *blaOXA-94*. L1, typically found in *S. maltophilia*, may contribute to cefiderocol resistance similarly to NDM.^34^ These data point to multifactorial resistance requiring further functional validation.

### Synergistic combinations including cefiderocol

Clinical trial outcomes of cefiderocol for treating *A. baumannii* infections have been mixed. The CREDIBLE-CR trial reported that patients with CRAB infections treated with cefiderocol demonstrated similar rates of infection resolution, but higher mortality rates compared to patients treated with the best available therapy, which usually included colistin-based regimens.^16^ However, this higher mortality rate may have been due to baseline differences in illness severity.^16^ Conversely, the APEKS-NP trial found that cefiderocol was non-inferior to meropenem for nosocomial pneumonia, although *A. baumannii* infections only comprised 16% of patients studied.^17^ With strong clinical data lacking, the IDSA recommends cefiderocol only when first-line options such as sulbactam/durlobactam are unavailable, and always in combination.^18^ However, few cefiderocol-containing antibiotic combinations effective against *A. baumannii* have been reported. One study demonstrated synergy between cefiderocol and fosfomycin against a CRAB isolate.^35^ Another recent study reported synergy between cefiderocol and either avibactam, sulbactam, meropenem, or amikacin against *A. baumannii* isolates.^36^ We found multiple antibiotics that demonstrate potent synergy when combined with cefiderocol.

Among the combinations tested, ceftazidime/avibactam and sulbactam/durlobactam demonstrated the most consistent and potent synergy with cefiderocol, with synergy observed in 100% and 95.2% of strains, respectively. These combinations significantly lowered cefiderocol MICs, including in strains with high baseline resistance. Broad-spectrum inhibition of classes A, C and D β-lactamases by avibactam and durlobactam likely protects β-lactam components from degradation, and additional activity against PBP3 likely enhances synergy.^37–40^ Synergy between cefiderocol and sulbactam/durlobactam is especially relevant, as sulbactam/durlobactam is a new β-lactam/β-lactamase inhibitor combination now recommended by the IDSA as first-line therapy for CRAB, after the ATTACK trial demonstrated sulbactam/durlobactam’s non-inferiority to colistin in treating CRAB infections.^18,26^ Sulbactam/durlobactam was highly effective against our collection, with 100.0% of strains susceptible (MIC ≤4/4).^24^ The combination of cefiderocol and either sulbactam/durlobactam or ceftazidime/avibactam may be a promising alternative for highly drug-resistant *A. baumannii*, and further clinical evaluation should be conducted.

Amikacin showed synergy in 66.7% of strains, including all cefiderocol-resistant ones, despite prevalent AMEs and *armA*.^21,41^ Though effective, aminoglycoside use must be weighed against toxicity risks.^42^ Doxycycline and cefiderocol also showed high synergy rates (61.9%), despite widespread TetB-mediated resistance.^21^ In contrast, omadacycline and eravacycline, though effective as monotherapies, showed limited synergy.^21^ Furthermore, no combinatorial effects with either minocycline or tigecycline during initial disk stacking screening were observed. Other non-β-lactam antibiotics showed variable efficacy. Rifampin showed modest efficacy, with synergy against 5 strains (23.8%) and additive effects against 9 strains (42.9%). While rifampin has shown *in vitro* synergy with colistin,^43^ clinical trials have not demonstrated improved outcomes.^44,45^ Ciprofloxacin also demonstrated modest synergy (19.0%) with cefiderocol, and additive effects against 42.9% of strains, despite universal resistance conferred by *gyrA* and *parC* mutations.^21,46^ Interactions between cefiderocol and non-β-lactam drugs may be due to improved drug uptake in the setting of cefiderocol-mediated membrane disruption.^47^

Other cephalosporins demonstrated variable synergy with cefiderocol. Synergy was observed with cefepime (47.6%), ceftriaxone (42.9%), ceftazidime (33.3%) and cefotaxime (23.8%). The differences in synergy among different cephalosporins may reflect differences in PBP3 targeting, although further research characterizing their exact mechanisms is needed. Sulbactam had higher synergy rates than ampicillin–sulbactam when combined with cefiderocol (52.4% versus 38.1%), suggesting the β-lactam component may not meaningfully enhance synergy with cefiderocol. Since ampicillin has a broad range of PBP targets in *Acinetobacter*,^48^ while both cefiderocol and sulbactam target PBP3,^11,38^ we expected increased synergy between cefiderocol and ampicillin/sulbactam due to increased PBP targets and additional β-lactamase protection from sulbactam. However, our findings suggest that cefiderocol and sulbactam alone were a more effective combination, warranting further investigation into the underlying mechanisms of their synergy. No antagonism was observed in any combination, suggesting that cefiderocol can be paired with a wide range of antibiotics. Overall, the most promising combinations, ceftazidime/avibactam, sulbactam/durlobactam, amikacin and doxycycline, demonstrated consistent synergy across numerous strains and are strong candidates for *in vivo* or clinical evaluation.

### Cefiderocol synergism against an MBL-producing strain

While sulbactam/durlobactam is now recommended as first-line therapy for CRAB, resistance has emerged, mainly due to NDM-type MBLs.^18,49,50^ MBL-producing *A. baumannii* pose a major challenge, as they also confer resistance to many other β-lactam drugs, including carbapenems and cefiderocol.^51,52^ MBLs are not inhibited by durlobactam and avibactam due to their zinc-based active site.^37,38,51^ None of the strains in our original collection (M1–M22) harbour MBLs, and all were susceptible to sulbactam/durlobactam.^21^ To evaluate activity against MBL-producing strains, we tested antibiotic combinations against BAA-3302, an MBL-carrying strain resistant to cefiderocol and non-susceptible to sulbactam/durlobactam. Cefiderocol with β-lactams or β-lactam/β-lactamase inhibitors, including ceftriaxone, cefotaxime, cefepime, ampicillin/sulbactam, ceftazidime/avibactam and sulbactam/durlobactam, showed strong synergy. Although MBLs reduce cefiderocol susceptibility, the drug is still relatively stable to MBL hydrolysis and has a weak binding affinity for the MBL active sites.^52^ This may allow cefiderocol to enhance the activity of other β-lactams to overcome MBL-mediated degradation.^52^ Additionally, the non-β-lactam drugs amikacin, ciprofloxacin and rifampin displayed strong synergy with cefiderocol against strain BAA-3302. This synergy may also be due to cefiderocol-mediated inhibition of peptidoglycan synthesis, which may compromise cell wall integrity, facilitating increased uptake of these agents.^47^ These findings suggest potential combination strategies for treating highly resistant, MBL-producing *A. baumannii*.

No synergy was observed between cefiderocol and tetracycline drugs. BAA-3302 was highly susceptible to tetracyclines, likely due to an absence of typical tetracycline resistance genes such as *tet(B)*. However, BAA-3302 possesses multiple RND efflux pumps not found in M1–M22, including *adeABC, adeFGH* and *adeIJK*, which extrude a wide range of antibiotics but have particularly high activity against tetracyclines.^53^ Active efflux of tetracyclines by RND pumps may have offset any increased membrane permeability conferred by cefiderocol, thus limiting intracellular tetracycline accumulation and preventing synergism.^53^ In contrast to BAA-3302, many strains in our M1–M22 collection exhibited synergy between cefiderocol and tetracyclines and other non-β-lactam antibiotics, including amikacin, ciprofloxacin and rifampin, possibly due to the absence of *adeABC, adeFGH* and *adeIJK*. A key limitation of this study is the inclusion of only a single MBL-harbouring strain. Future studies should evaluate a broader panel of MBL-producing isolates with diverse resistance gene profiles to better characterize cefiderocol-based combination efficacy.

## Conclusions

In this study, we evaluated the *in vitro* activity of cefiderocol against XDR and PDR *A. baumannii* and identified synergistic cefiderocol-based combinations. cefiderocol alone was active against 66.7% of strains, and combinations with ceftazidime/avibactam and sulbactam/durlobactam showed synergy in 100% and 95.7%, respectively. Other effective antibiotics in combination with cefiderocol were amikacin, doxycycline and sulbactam. We also identified cefiderocol-containing combinations effective against an *NDM-*1-harbouring *A. baumannii* strain resistant to cefiderocol and highlighted potential mechanisms underlying cefiderocol resistance and synergy patterns. Our findings may help guide decision-making in selecting additional antibiotics to combine with cefiderocol in the treatment of highly drug-resistant *A. baumannii* infections.

## Supplementary Material

dkaf306_Supplementary_Data

## Supplementary data

Figures S1 to S10 and Tables S1 and S2 are available as Supplementary data at *JAC* Online.
