# Supplementary material for: Synergistic cefiderocol-containing antibiotic combinations active against highly drug-resistant Acinetobacter baumannii patient isolates with diverse resistance mechanisms
Source: J Antimicrob Chemother. 2025 Aug 20;80(10):2814–24. doi: 10.1093/jac/dkaf306 (PMC12494139; doi:10.1093/jac/dkaf306)

Supporting Information

**Cefiderocol-containing antibiotic combinations effective against extensively- and pandrug-resistant *Acinetobacter baumannii* patient isolates**

Justin Halim, Andrew P. Keane, Jeannete Bouzo, Tope Aderibigbe, Jessica A. Chicola, Katie T. Nolan, Keertana Jonnalagadda, Jason X. Tran, and Valerie J. Carabetta

Table of Contents

[Figure S1: Representative checkerboard assay with cefiderocol and eravacycline against strain M5. 2](#_Toc203461307)

[Figure S2: Representative checkerboard assay with cefiderocol and omadacycline against strain M12. 3](#_Toc203461308)

[Figure S3: Representative checkerboard assay with cefiderocol and rifampin against strain M4. 4](#_Toc203461309)

[Figure S4: Representative checkerboard assay with cefiderocol and ciprofloxacin against strain M4. 5](#_Toc203461310)

[Figure S5: Representative checkerboard assay with cefiderocol and ceftazidime against strain M7. 6](#_Toc203461311)

[Figure S6: Representative checkerboard assay with cefiderocol and cefepime against strain M22. 7](#_Toc203461312)

[Figure S7: Representative checkerboard assay with cefiderocol and ceftriaxone against strain M13. 8](#_Toc203461313)

[Figure S8: Representative checkerboard assay with cefiderocol and cefotaxime against strain M7. 9](#_Toc203461314)

[Figure S9: Representative checkerboard assay with cefiderocol and ampicillin/sulbactam against strain M3. 10](#_Toc203461315)

[Figure S10: Representative checkerboard assay with cefiderocol and sulbactam against strain M3. 11](#_Toc203461316)

[Figure S11: Representative checkerboard assay with cefiderocol and ciprofloxacin against BAA-3302. 12](#_Toc203461317)

[Table S1: Results of the disc stacking synergy screening. 13](#_Toc203461318)

[Table S2: Comparison of sulbactam/durlobactam MIC values in MHB, CAMHB, and ID-CAMHB. 14](#_Toc203461319)

# **Figure S1:** Representative checkerboard assay with cefiderocol and eravacycline against strain M5.

Top: OD_600_ measurements following 16 hours of static growth at 37°C. The MIC values for eravacycline and cefiderocol alone are highlighted in gray. The pink boxes indicate wells in which no bacterial growth occurred (OD_600_ <0.1), and green boxes indicate wells in which bacterial growth did occur. Bottom: Fractional inhibitory concentration (FIC) values were calculated for each drug (concentration/MIC) and added together for all wells where no growth was observed. The yellow boxes indicate additive interactions (FICI between 0.5-1.0), and blue boxes indicate synergistic interactions (FICI ≤0.5).

# **Figure S2:** Representative checkerboard assay with cefiderocol and omadacycline against strain M12.

Top: OD_600_ measurements following 16 hours of static growth at 37°C. The MIC values for cefiderocol and omadacycline alone are highlighted in gray. The pink boxes indicate wells in which no bacterial growth occurred (OD_600_ <0.1), and green boxes indicate wells in which bacterial growth did occur. Bottom: Fractional inhibitory concentration (FIC) values were calculated for each drug (concentration/MIC) and added together for all wells where no growth was observed. The yellow boxes indicate additive interactions (FICI between 0.5-1.0), and blue boxes indicate synergistic interactions (FICI ≤0.5).

# **Figure S3:** Representative checkerboard assay with cefiderocol and rifampin against strain M4.

Top: OD_600_ measurements following 16 hours of static growth at 37°C. The MIC value for cefiderocol alone is highlighted in gray. There is no highlighted MIC value for rifampin as the MIC exceeded the maximum starting concentration. The pink boxes indicate wells in which no bacterial growth occurred (OD_600_ <0.1), and green boxes indicate wells in which bacterial growth did occur. Bottom: Fractional inhibitory concentration (FIC) values were calculated for each drug (concentration/MIC) and added together for all wells where no growth was observed. The yellow boxes indicate additive interactions (FICI between 0.5-1.0), and blue boxes indicate synergistic interactions (FICI ≤0.5).

# **Figure S4:** Representative checkerboard assay with cefiderocol and ciprofloxacin against strain M4.

Top: OD_600_ measurements following 16 hours of static growth at 37°C. The MIC values for cefiderocol and ciprofloxacin alone are highlighted in gray. The pink boxes indicate wells in which no bacterial growth occurred (OD_600_ <0.1), and green boxes indicate wells in which bacterial growth did occur. Bottom: Fractional inhibitory concentration (FIC) values were calculated for each drug (concentration/MIC) and added together for all wells where no growth was observed. The yellow boxes indicate additive interactions (FICI between 0.5-1.0), and blue boxes indicate synergistic interactions (FICI ≤0.5).

# **Figure S5:** Representative checkerboard assay with cefiderocol and ceftazidime against strain M7.

Top: OD_600_ measurements following 16 hours of static growth at 37°C. The MIC values for ceftazidime and cefiderocol alone are highlighted in gray. The pink boxes indicate wells in which no bacterial growth occurred (OD_600_ <0.1), and green boxes indicate wells in which bacterial growth did occur. Bottom: Fractional inhibitory concentration (FIC) values were calculated for each drug (concentration/MIC) and added together for all wells where no growth was observed. The yellow boxes indicate additive interactions (FICI between 0.5-1.0), and blue boxes indicate synergistic interactions (FICI ≤0.5).

# **Figure S6:** Representative checkerboard assay with cefiderocol and cefepime against strain M22.

Top: OD_600_ measurements following 16 hours of static growth at 37°C. The MIC values for cefepime and cefiderocol alone are highlighted in gray. The pink boxes indicate wells in which no bacterial growth occurred (OD_600_ <0.1), and green boxes indicate wells in which bacterial growth did occur. Bottom: Fractional inhibitory concentration (FIC) values were calculated for each drug (concentration/MIC) and added together for all wells where no growth was observed. The yellow boxes indicate additive interactions (FICI between 0.5-1.0), and blue boxes indicate synergistic interactions (FICI ≤0.5).

# **Figure S7:** Representative checkerboard assay with cefiderocol and ceftriaxone against strain M13.

Top: OD_600_ measurements following 16 hours of static growth at 37°C. The MIC values for ceftriaxone and cefiderocol alone are highlighted in gray. The pink boxes indicate wells in which no bacterial growth occurred (OD_600_ <0.1), and green boxes indicate wells in which bacterial growth did occur. Bottom: Fractional inhibitory concentration (FIC) values were calculated for each drug (concentration/MIC) and added together for all wells where no growth was observed. The yellow boxes indicate additive interactions (FICI between 0.5-1.0), and blue boxes indicate synergistic interactions (FICI ≤0.5).

# **Figure S8:** Representative checkerboard assay with cefiderocol and cefotaxime against strain M7.

Top: OD_600_ measurements following 16 hours of static growth at 37°C. The MIC value for cefiderocol alone is highlighted in gray. There is no highlighted MIC value for cefotaxime as the MIC exceeded the maximum starting concentration. The pink boxes indicate wells in which no bacterial growth occurred (OD_600_ <0.1), and green boxes indicate wells in which bacterial growth did occur. Bottom: Fractional inhibitory concentration (FIC) values were calculated for each drug (concentration/MIC) and added together for all wells where no growth was observed. The yellow boxes indicate additive interactions (FICI between 0.5-1.0), and blue boxes indicate synergistic interactions (FICI ≤0.5).

# **Figure S9:** Representative checkerboard assay with cefiderocol and ampicillin/sulbactam against strain M3.


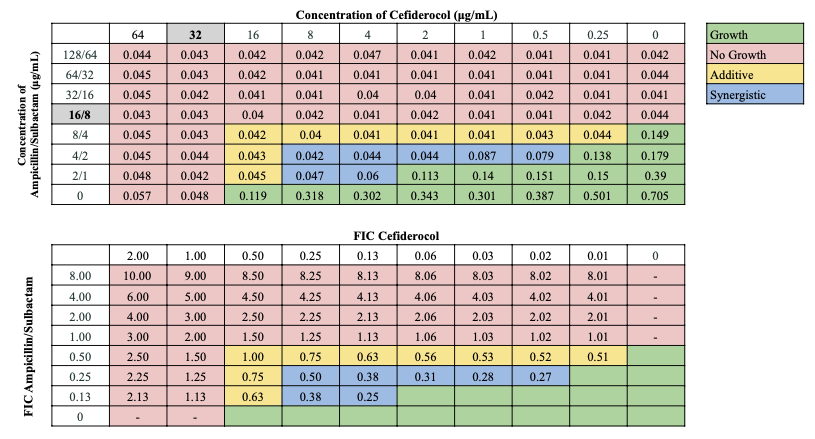


Top: OD_600_ measurements following 16 hours of static growth at 37°C. The MIC values for each drug alone are highlighted in gray. The pink boxes indicate wells in which no bacterial growth occurred (OD_600_ <0.1), and green boxes indicate wells in which bacterial growth did occur. Bottom: FIC values were calculated for each drug (concentration/MIC) and added together for all wells where no growth was observed. The yellow boxes indicate additive interactions (FICI between 0.5-1.0), and blue boxes indicate synergistic interactions (FICI ≤0.5).

# **Figure S10:** Representative checkerboard assay with cefiderocol and sulbactam against strain M3.


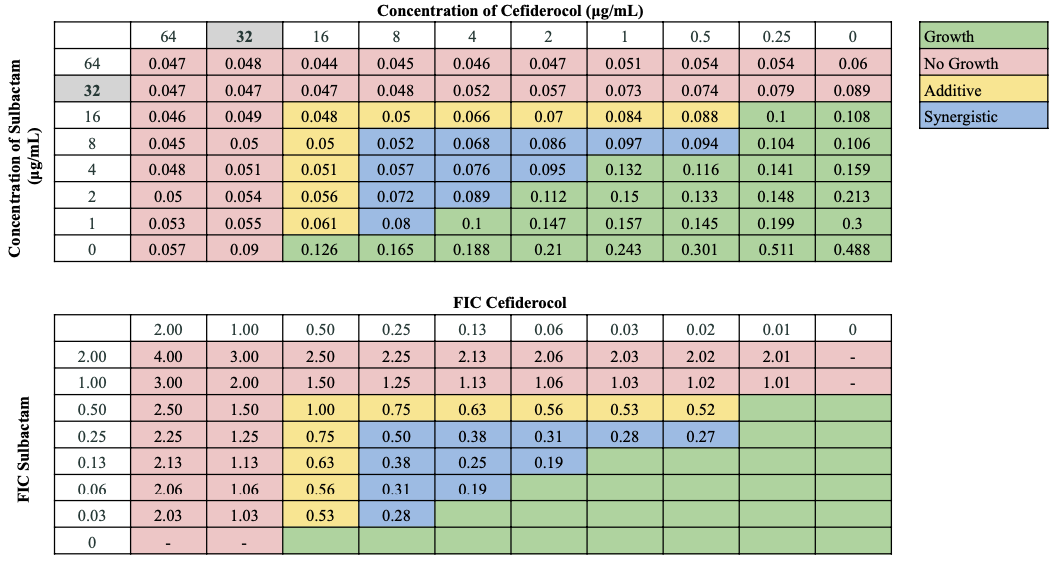


Top: OD_600_ measurements following 16 hours of static growth at 37°C. The MIC values for each drug alone are highlighted in gray. The pink boxes indicate wells in which no bacterial growth occurred (OD_600_ <0.1), and green boxes indicate wells in which bacterial growth did occur. Bottom: FIC values were calculated for each drug (concentration/MIC) and added together for all wells where no growth was observed. The yellow boxes indicate additive interactions (FICI between 0.5-1.0), and blue boxes indicate synergistic interactions (FICI ≤0.5).

# **Figure S11:** Representative checkerboard assay with cefiderocol and ciprofloxacin against BAA-3302.


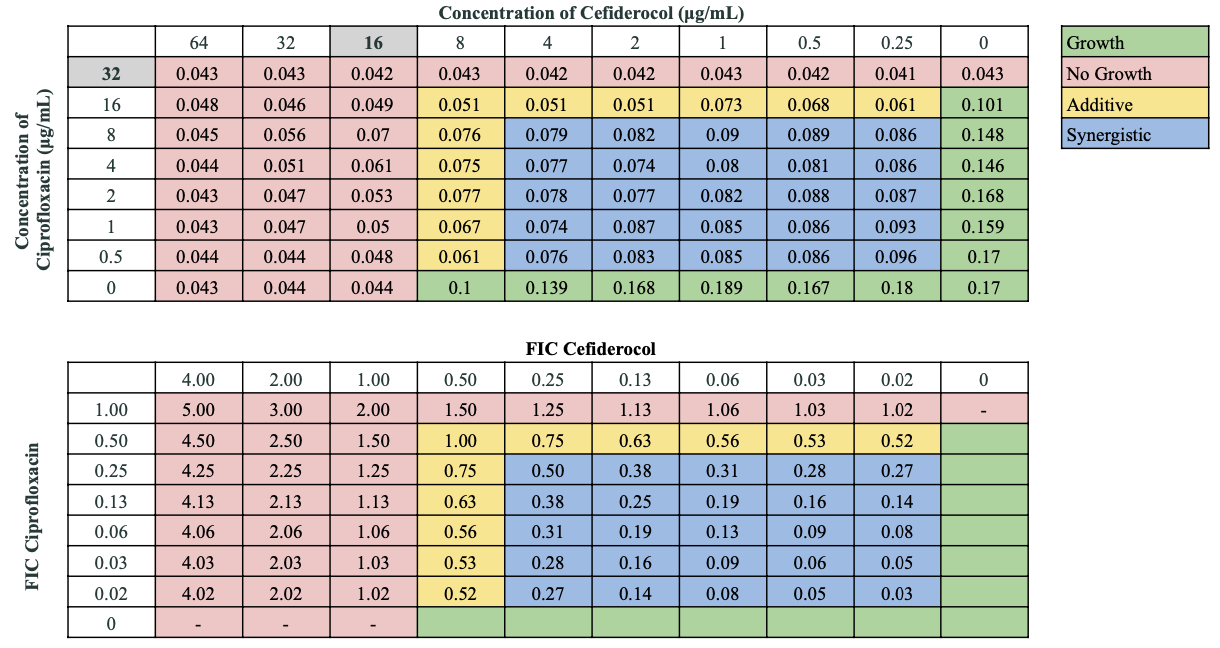


Top: OD_600_ measurements following 16 hours of static growth at 37°C. The MIC value for cefiderocol alone is highlighted in gray. There is no highlighted MIC value for cefotaxime as the MIC exceeded the maximum starting concentration. The pink boxes indicate wells in which no bacterial growth occurred (OD_600_ <0.1), and green boxes indicate wells in which bacterial growth did occur. Bottom: Fractional inhibitory concentration (FIC) values were calculated for each drug (concentration/MIC) and added together for all wells where no growth was observed. The yellow boxes indicate additive interactions (FICI between 0.5-1.0), and blue boxes indicate synergistic interactions (FICI ≤0.5).

# **Table S1:** Results of the disc stacking synergy screening.

| \| **Paired Antibiotic** \| **Interaction Visualized** \| \| --- \| --- \| \| Meropenem \| No \| \| Cefepime \| Yes \| \| Ceftriaxone \| Yes \| \| Cefotaxime \| Yes \| \| Ceftazidime \| Yes \| \| Ceftazidime/Avibactam \| Yes \| \| Piperacillin/Tazobactam \| No \| \| Ampicillin/Sulbactam \| Yes \| \| Sulbactam \| Yes \| \| Doxycycline \| Yes \| \| Minocycline \| No \| \| Tigecycline \| No \| \| Tobramycin \| No \| \| Amikacin \| Yes \| \| Levofloxacin \| No \| \| Ciprofloxacin \| Yes \| \| Rifampin \| Yes \| |
| --- | --- | --- | --- | --- | --- | --- | --- | --- | --- | --- | --- | --- | --- | --- | --- | --- | --- | --- | --- | --- | --- | --- | --- | --- | --- | --- | --- | --- | --- | --- | --- | --- | --- | --- | --- | --- |

* Strain M9 was used for screening assays.

# **Table S2:** Comparison of sulbactam/durlobactam MIC values in MHB, CAMHB, and ID-CAMHB.


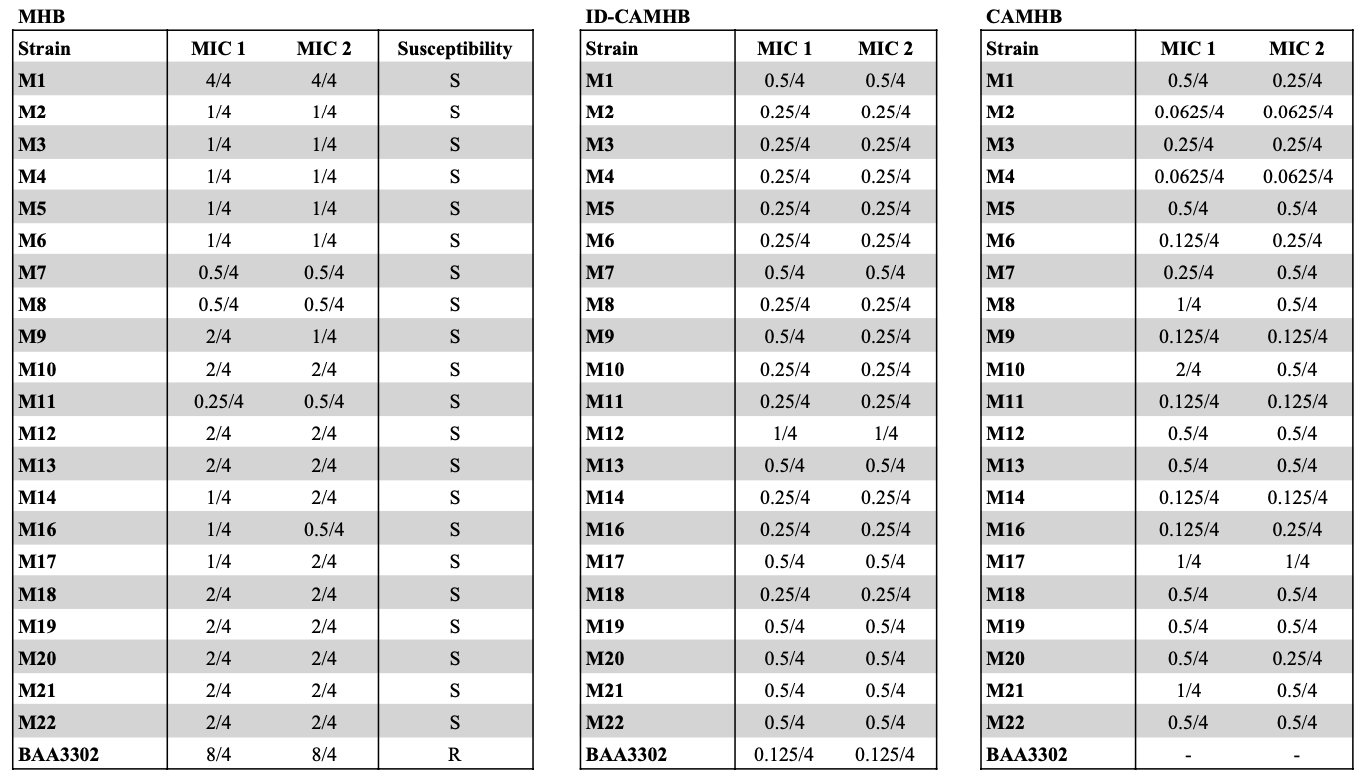

Supplement: dkaf306_Supplementary_Data [file dkaf306_supplementary_data.docx]
